# Understanding tumor localization in multiparametric MRI of the prostate—effectiveness of 3D printed models

**DOI:** 10.3389/fsurg.2023.1264164

**Published:** 2023-09-20

**Authors:** Maximilian Haack, Katja Reisen, Ahmed Ghazy, Kristina Stroh, Lisa Frey, Peter Sparwasser, Gregor Duwe, Rene Mager, Axel Haferkamp, Hendrik Borgmann

**Affiliations:** ^1^ Department of Urology and Pediatric Urology, Johannes-Gutenberg-University Medical Center, Mainz, Germany; ^2^ Department of Cardiovascular Surgery, Johannes-Gutenberg-University Medical Center, Mainz, Germany; ^3^ Department of Diagnostic and Interventional Radiology, Johannes-Gutenberg-University Medical Center, Mainz, Germany; ^4^ Department of Urology, Faculty of Health Sciences Brandenburg, Brandenburg Medical School Theodor Fontane, Brandenburg an der Havel, Germany

**Keywords:** prostate, prostate carcinoma, prostate biopsy, MRI of the prostate, PI-RADS, 3D printed prostate model

## Abstract

**Introduction:**

Understanding tumor localization in multiparametric MRI (mpMRI) of the prostate is challenging for urologists but of great importance in mpMRI-fused prostate biopsy or radical prostatectomy. The aim was to evaluate the effectiveness of 3D printed models of the prostate to help urologists to locate tumors.

**Methods and Participants:**

20 urologists from University Medical Center Mainz (Germany) were asked to plot the location of a cancer suspicious lesion (PI-RADS ≥ 4) on a total of 30 mpMRI on a prostate sector diagram. The following 3 groups (as matched triplets) were divided into: mpMRI only, mpMRI with radiological report and mpMRI with 3D printed model (scaled 1:1). Statistical analysis was performed using one-way and two-way ANOVA (with bonferroni post-test).

**Results:**

Overall, localization of the suspicious lesion was superior with the radiological report (median of max. 10 [IQR]: MRI 2 [IQR 1;5], MRI + report: 8 [6.3;9], MRI + 3D model 3 [1.3;5.8]; *p* < 0.001). Residents with <1 year of experience had a significantly higher detection rate using a 3D printed model [5 (5;5.8)] compared to mpMRI alone [1.5 (1;3.5)] (*p* < 0.05). Regarding the estimation of index lesion extension, the 3D model showed a significant benefit (mean percentage difference [95% CI]: MRI alone 234% [17.1;451.5], MRI + report 114% [78.5;149.6], MRI + 3D model 17% [−7.4;41.3] (*p* < 0.01).

**Conclusion:**

Urologists still need the written radiological report for a sufficient understanding of tumor localization. The effectiveness of the 3D printed model regarding tumor localization is particularly evident in young residents (<1 year) and leads to a better overall assessment of the tumor extension.

## Introduction

Multiparametric Magnetic Resonance Imaging (mpMRI) of the prostate has become a standard diagnostic method for patients with suspected prostate cancer. The resulting mpMRI targeted prostate biopsy significantly increased the detection rate of clinically significant prostate cancer (1, 2). Therefore, mpMRI of the prostate has been implemented in several urological guidelines as mandatory diagnostic before prostate biopsy (2, 3). However, understanding mpMRI regarding tumor localization remains a major challenge for urologist and even radiologists worldwide (4–6). Physician's experience with mpMRI has shown to be an important factor for the reliable reporting of mpMRIs (7, 8). This is also reflected in a prolonged learning curve for reliably performing mpMRI targeted prostate biopsies (9).

Since these procedures are usually performed by residents it is crucial that they can gain a comprehensive understanding of mpMRI of the prostate quickly to ensure a sufficient detection rate of the prostate biopsy. This is particularly crucial for the indication of nerve sparing in the context of radical prostatectomy, as the quality of the preoperative biopsy represents a significant risk factor for a positive surgical margin (10). The use of printed three-dimensional (3D) prostate models or virtual 3D models of the prostate for surgical planning or education has been evaluated in several studies (7, 11, 12). It has shown to improve physicians' orientation and localization of suspicious lesions in mpMRI (7, 11) and can help with patient education as well (13).

Our study aimed to investigate the effectiveness of 3D printed prostate models regarding tumor localization in mpMRI. Furthermore, the study focuses on the impact of the 3D model on different levels of experience of urologists.

## Methods and participants

### Study design and population

A total of 20 urologists of different levels of experience (4 residents <1 years, 4 residents >3 years, 8 specialists >6 years and 4 senior specialists >10 years) were reviewed in this single-center, prospective study from June 2022 to December 2022. Each participant was asked to locate a singular suspicious lesion in the prostate-mpMRI of 30 cases in total. The localization was carried out by marking the lesion on the prostate sector diagram used by the European society of Urogenital Radiology and American College of Radiology (14). The 30 cases were divided into three equally sized groups as matched triplets. The first group included only the mpMRI sequences, so that the physician had no further information. The second group represented the clinical standard with mpMRI-sequences and radiological report. In the third group mpMRI-sequences were supplemented by a corresponding printed 3D model of the prostate (scaled 1:1) where the tumor was highlighted with red color. The duration of the survey was recorded with a stopwatch. At the end, each participant received a 10-item questionnaire (5-point Likert scale; 1: very poor; 2: poor; 3: fair; 4: good; 5: excellent) that asked about the perceived usefulness of the 3D model and the perceived certainty in mpMRI reporting.

### mpMRI of the prostate

All mpMRI sequences were realized using 3-Tesla mpMRI at our center, which included T1-weighted imaging (T1WI), T2-weighted imaging (T2WI), diffusion weighted imaging (DWI) and dynamic contrast-enhanced MR imaging (DCE-MRI). A special T2WI sequence with 1 mm layer thickness was created for a more seamless printing of the 3D prostate models. Assessment of the mpMRI studies was performed according to the Prostate Imaging and Reporting Data System (PI-RADS) version 2.1. Only singular target lesions found with PI-RADS 4 or 5 were used for this study. In each of the three groups, we collected 7 cases with PI-RADS 4 and 3 cases with PI-RADS 5. There was no limitation in target lesion size. The target lesion size ranged from 3 to 408 mm^2^. Same side, level, zone and PI-RADS score of the lesion, as well as similar prostate size were used to create matched triplets. Cases with multiple lesions and a prostate volume of >100 ml were excluded.

### Printed 3D models

**Figure 1:**
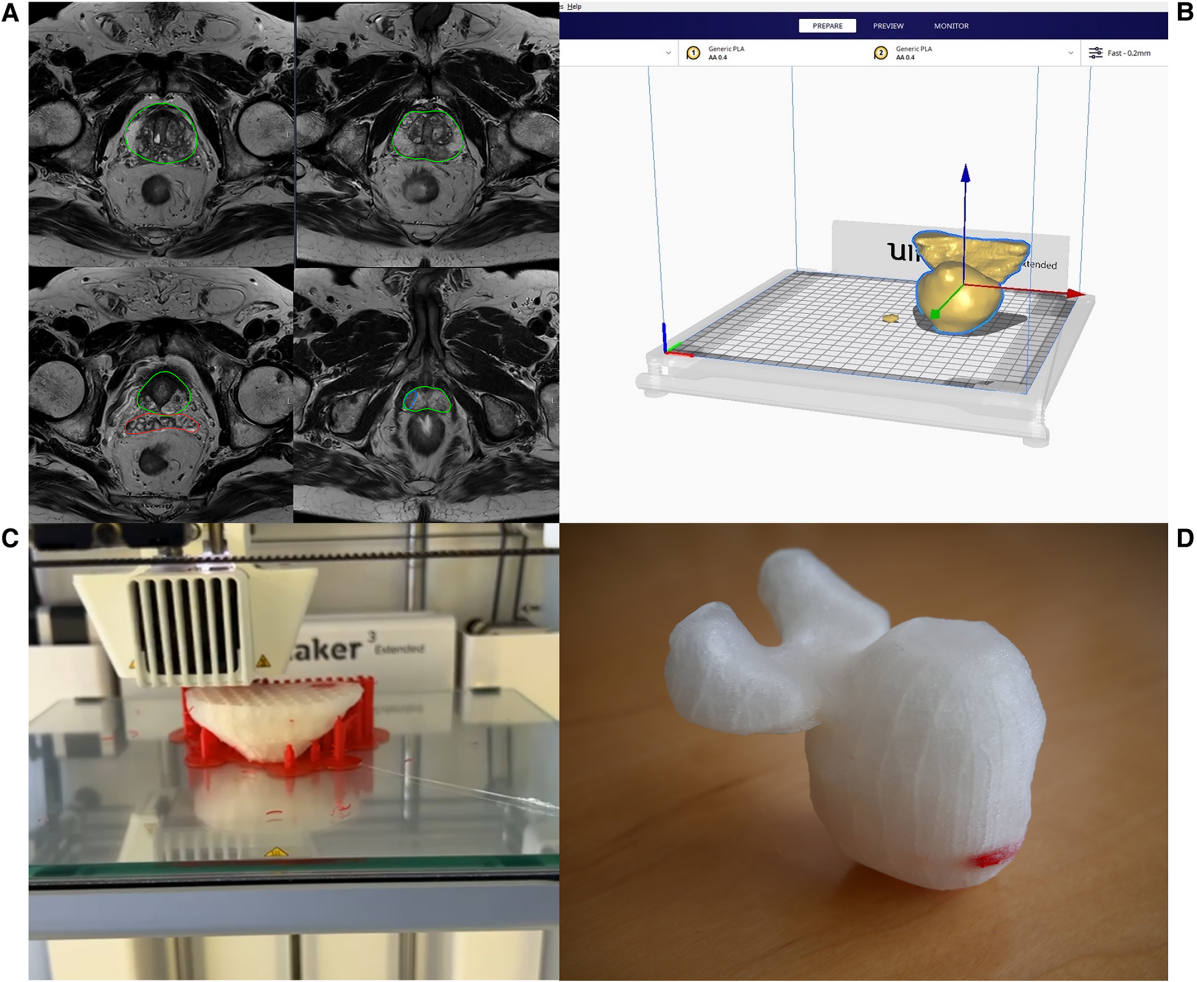
Prostate segmentation from mpMRI series (**A**), digitalizing the 3D model (**B**), 3D printing (**C**), finished 3D printed prostate model with the tumor indicated in bright red color (**D**).

Segmentation of the 3D prostate models was realized with the DICOM files (Digital Imaging and Communications in Medicine) of the mpMRI sequences with 1 mm layer thickness using Materialise Mimics® (Version 24.0.0.427) and 3-matic® (Version 16.0) (Materialise NV, Leuven, Belgium). The digitalized 3D model was then exported and printed using the UltiMaker® 3 Extended with dual extruder (released 2015, Ultimaker®, Utrecht, Netherlands). Prostate and both seminal bladders were printed continuously with a transparent polylactic acid filament (PLA), whereas the tumor was printed into the prostate using a PLA-filament in bright red color (Figure 1).

### Data acquisition

**Figure 2:**
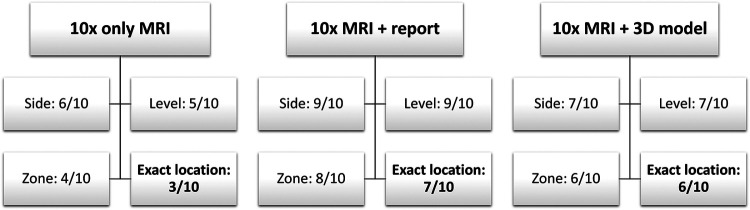
Exemplary scoring of primary outcomes (side, level, zone and exact location) of one participant in all 3 groups (only MRI, MRI + report and MRI + 3D model).

**Figure 3:**
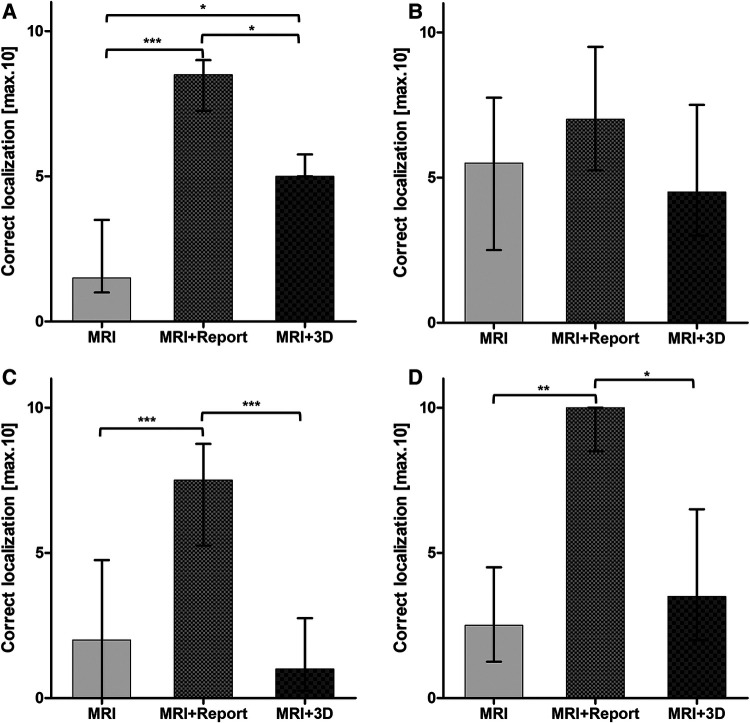
Correct localization of the index lesion in all three modalities differentiated into four groups of experience: (**A**) <1 year residents; (**B**) >3 year residents; (**C**) >6 year specialists; (**D**) >10 year senior specialists. Statistical significance was *p* < 0.05.

For the interviews, the mpMRI sequences were sorted alphabetically by patient name. Thus, the matched triplets were not comprehensible for the participant. For data analysis, the cases were reorganized according to the matched triplets so that a comparison was possible. Primary Outcomes were side (left or right), level (basis, midgland, apex), zone (e.g., lateral peripheral) and exact location of the lesion (if all previous outcome items were correct). Location of the index lesion was marked on the prostate sector diagram (14). The score for each outcome item was binary (correct or false), so that the maximum score for each outcome item was 10. The scoring system is further explained in Figure 2. Secondary Outcome was the duration of the interview as well as the percentage deviation of the area of the lesion, but only in those cases where the exact location was correct. The area extension was measured from the markings on the prostate sector diagram (14) by of the participant and compared with the radiologist's markings (Figure 3).

### Statistical analysis

Statistical analysis was performed using one-way and two-way ANOVA (with bonferroni post-test). All data were analyzed using GraphPad PRISM® 5 (Version 5.01, 2007, GraphPad Software Inc., Boston, USA). Statistical significance was defined as *p* < 0.05.

## Results

### Tumor localization and extension

Overall, tumor localization was superior in all primary outcome items when using mpMRI and radiological report: side [6 (5;7) vs. 10 (10;10) vs. 8 (6.3;10); *p* < 0.001], level [4 (2.3;6) vs. 10 (9;10) vs. 5 (4;6.8); *p* < 0.001], zone [5 (4;6.8) vs. 8 (7;9) vs. 5.5 (3;8); *p* < 0.001] and exact location [2 (1;5) vs. 8 (6.3;9) vs. 3 (1.3;5.8); *p* < 0.001] (Table 1). The most correct localization was achieved by the >10-year senior specialists with the additional use of the radiological findings (Figure 3). The >3-year residents showed no significant difference between all 3 groups and performed best when given only the mpMRI sequences (Table 1; Figure 3). A significant benefit of the 3D prostate model compared with mpMRI reporting alone could be shown within the <1-year residents [5. (5;5.8) vs. 1.5 (1;3.5); *p* < 0.05] (Figure 3).

**Table 1:** Localizations and deviation of the lesion extension in the three modalities.

*n* = 20	MRI		MRI + report		MRI + 3D-model	
	Median [IQR]	Mean [95% CI]	Median [IQR]	Mean [95% CI]	Median [IQR]	Mean [95% CI]
Side	6 [5;7]		10 [10;10]		8 [6.3;10]	
Level	4 [2.3;6]		10 [9;10]		5 [4;6.8]	
Zone	5 [4;6.8]		8 [7;9]		5.5 [3;8]	
Exact location	2 [1;5]		8 [6.3;9]		3 [1.3;5.8]	
<1 year (*n* = 4)	1.5 [1;3.5]		8.5 [7.3;9]		5 [5;5.8]	
>3 year (*n* = 4)	5.5 [2.5;7.8]		7 [5.3;9.5]		4.5 [3;7.5]	
>6 year (*n* = 8)	2 [0;4.8]		7.5 [5.3;8.8]		1 [0.3;2.8]	
>10 year (*n* = 4)	2.5 [1.3;4.5]		10 [8.5;10]		3.5 [2;6.5]	
Area deviation [%]		234 [17.1;451.5]		114 [78.5;149.6]		17 [−7.4;41.3]

**Figure 4:**
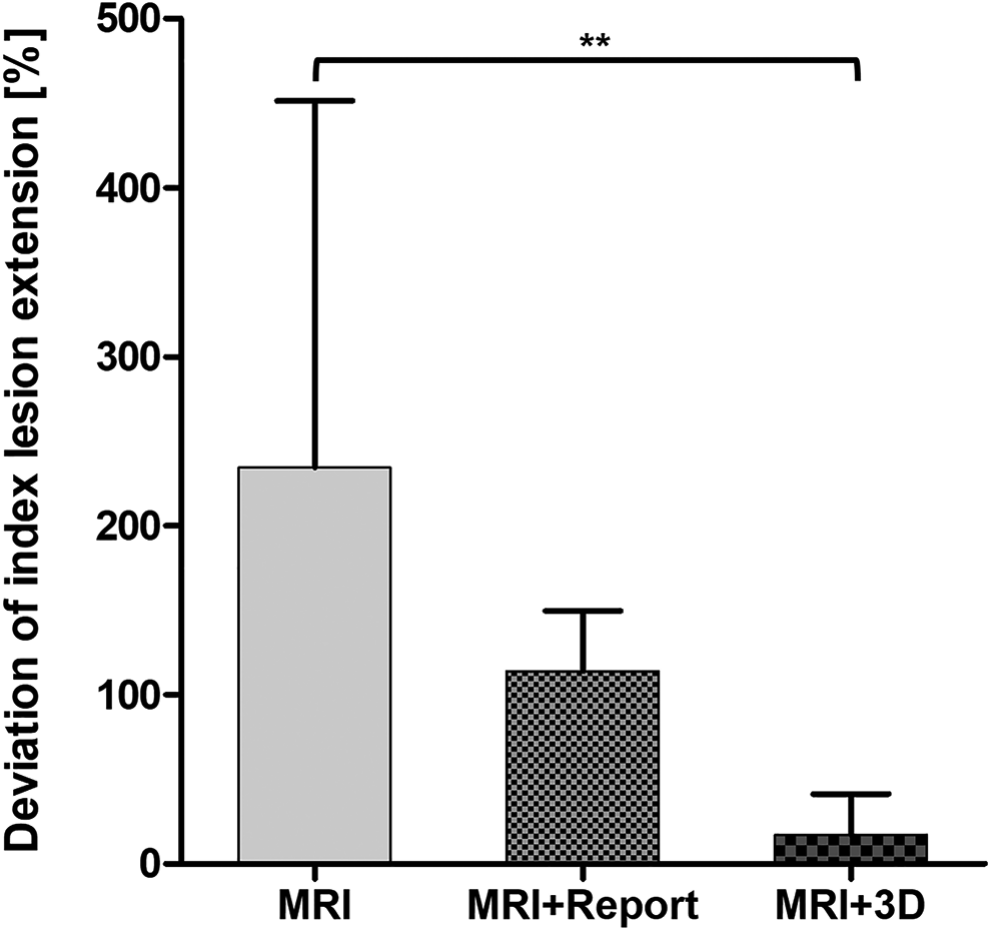
Deviation of index lesion extension marked on the prostate sector diagram. Statistical significance was *p* < 0.05.

Participants overestimated the lesion extension by a mean of 234% with mpMRI alone. The overestimation decreased to 114% with the radiological report. The most accurate assessment of the tumor extension was achieved with the 3D model (17%) (Figure 4).

Mean survey duration was 47.7 min [38.9;56.6]. Fastest completion time with 31.7 min on average [27.9;35.5] was recorded in the >3-year residents. Unsurprisingly, the <1-year residents took the longest time to complete the survey [58.6 (19.2;97.8)]. However, the times did not differ significantly between the experience categories (*p* = 0.394).

### Perceived usefulness and certainty in reporting

Most participants found the use of the 3D model in locating the lesion very useful [4.4 (4;4.8)] (Table 2). However, estimated benefits of the 3D prostate model on pre- or intraoperative usage regarding radical prostatectomy was moderate [3.6 (3.1;4.1) vs. 3.9 (3.4;4.3)]. The 3D prostate model was perceived to be useful regarding training of inexperienced surgeons or biopseurs as well as patient education [4.6 (4.3;4.9) and 4 (3.4;4.6)]. The certainty in localizing the index lesion was the same using the radiological report and the 3D prostate model [4.2 (3.9;4.5) vs. 4.2 (3.7;4.6)]. However, the uncertainty in the localization increased when only the mpMRI was available [2.5 (2;3.1)].

**Table 2:** Questionnaire for perceived usefulness and certainty in reporting on a 5-point Likert scale (1: very poor; 2: poor; 3: fair; 4: good; 5: excellent).

Number	Question	Mean [95% CI]
1	How helpful do you find the use of the 3D model in locating the index lesion?	4.4 [4;4.8]
2	How well do you think the 3D model reflects the anatomical conditions?	4.5 [4.2;4.9]
3	How great do you see the benefit of the 3D model in preoperative planning (e.g. in relation to nerve sparing)?	3.6 [3.1;4.1]
4	How useful do you think the 3D model is intraoperatively?	3.9 [3.4;4.3]
5	How well do you think the 3D model can be implemented in the training of inexperienced surgeons/biopseurs?	4.6 [4.3;4.9]
6	How useful do you consider the 3D model in terms of patient education and preoperative preparation?	4 [3.4;4.6]
7	How high do you see the benefit of the 3D model in terms of time efficiency?	3.7 [3.1;4.2]
8	How confident do you feel in locating the index lesion with radiological findings and MRI sequences?	4.2 [3.9;4.5]
9	How confident do you feel in locating the index lesion with 3D-prostate model and MRI sequences?	4.2 [3.7;4.6]
10	How confident do you feel in locating the index lesion using only the MRI sequences?	2.5 [2;3.1]

## Discussion

A precise knowledge of the tumor localization in mpMRI is mandatory for an accurate targeted prostate biopsy. In many cases, the biopseur has to transmit the region of interest onto the ultrasonic image for a mpMRI-Ultrasound fusion biopsy or perform a cognitive targeted biopsy (9, 15). Even in radical prostatectomy, a detailed understanding of the tumor localization in mpMRI as well as a high quality prostate biopsy is essential for safe resection margins and nerve sparing (10, 16). However, achieving a comprehensive understanding of mpMRI is still a major challenge for many urologists worldwide (4, 5) and requires a high level of experience (4, 7, 9, 17). After implementation of mpMRI and targeted prostate biopsies into the EAU-guidelines as the gold standard diagnostic for prostate cancer (3) the demand for targeted prostate biopsies has increased drastically. With a progressing shortfall of healthcare workers relative to population growth the number of highly trained urologists is declining as well (18–21). This effect is especially aggravating in rural areas, as specialists tend to gravitate towards urban settings (20). The divergence in demand and resources could lead to inexperienced urologists performing targeted prostate biopsies. To address this issue, technologies have evolved to help with three-dimensional orientation, planning and education, such as printed 3D models and virtual reality (7, 11, 22). The effectiveness of these tools has been demonstrated in several studies before, but there is still too little data to justify a more widespread use.

Therefore, the aim of this study was to provide more data on the effectiveness of 3D printed models of the prostate and focus on the impact on different levels of urologists' experience. Consistent with some previous findings (4), urologists of different levels of experience were found to require written radiological findings for reliable interpretation of mpMRIs (Table 1). Interestingly residents with >3 years of experience performed best between all groups when interpreting mpMRI only with the mpMRI-sequences (Table 1). This result could be explained by the fact that biopsies are performed most frequently at this level of training in our clinic. As Lee and Mager et al. have already shown, there is a learning curve of 40-50 targeted biopsies, after which the detection rate is sufficient and sometimes even higher than the expert standard of the institution (9, 17). This is likely a practice effect, which makes those who perform many biopsies at the time of the survey perform better. This is also supported by the short duration time to complete the interview of the >3-year residents. Interestingly there was a significant benefit of the printed 3D model within the <1-year residents (Table 1). This underlines the great usefulness of the 3D model in three-dimensional orientation since they have not had much exposure to mpMRI imaging in their career. Therefore, this result is consistent with the results from other studies (7, 23). Surprisingly, the >6-year specialists performed worse when using the printed 3D model compared to no additional aids (Table 1). This remains unclear and requires further investigation. As expected, the senior specialists (>10 years of experience) performed best overall when using the radiological reports (Table 1). The impact of the 3D model was similar compared to the >3-year residents but we could not detect a significant difference to the sole mpMRI interpretation as was only within the <1-years residents (Figure 3). The assessment of index lesion extension was significantly decreased when using the 3D model (Figure 4). Interestingly the participants overestimated the area in all three groups (Figure 4). However, the variance was greatest when only the mpMRI sequences were available. This reflects the limited experience with mpMRI assessment that still exists among many urologists (4). This emphasizes a great need for more training and visual aids. The overall uncertainty of the participants in interpreting mpMRI sequences when no other tools are available supports this even more (Table 2). The printed 3D model was perceived to be very useful in terms of anatomical resemblance, three-dimensional orientation and localizing, training and patient education (Table 2). However, it was rated only moderately useful in preoperative and intraoperative use (Table 2). This contradicts with some other studies, where the pre- and intraoperative utility of the 3D prostate model in prostate-specific surgery was considered to be useful (12, 24). 3D printed prostate models could also improve accuracy of targeted biopsies of PSMA-positive areas within the prostate since biopsy fusion software is mostly designed for mpMRI and post radiation effects reduce accuracy of mpMRI (25). Hereby, diagnostic certainty of PSMA-PET-CT for prostate cancer recurrence after curative prostate radiation could be improved.

In summary, our study provides significant data that supports the effectiveness of printed 3D models of the prostate in the localization of the tumor in mpMRI, especially with inexperienced urological residents (<1 year). However, urologists still need radiological reports to sufficiently locate the index lesion in mpMRI. Furthermore, our study showed a significant benefit of the printed 3D model of the prostate regarding assessment of the extension of the index lesion. The utility of the 3D model was considered useful regarding spatial orientation, training of inexperienced physicians and patient education. With the help of technological advances and more accessible 3D visualization tools the challenge of interpreting mpMRI of the prostate could become less in the future and improve prostate cancer diagnostics as well as patient care.

Our study also has some limitations. Study population was relatively small, especially in each of the four experience-categories. The effect of the 3D prostate model could be further evaluated in a multicentered follow-up-study. Furthermore, we only included singular index lesions with a PI-RADS score ≥4. To evaluate the impact of the 3D model in a more realistic scenario multiple lesions and PI-RADS 3 lesions would need to be included. Moreover, the transparent PLA-filament used for prostate and seminal bladders turned out to be partially transparent after printing. Hereby, lesions that were located more central were hard to see from the outside. As a solution, an acrylic filament could be used for complete transparency.

## Conclusion

Understanding tumor localization in multiparametric MRI of the prostate still requires written radiological reports for sufficient interpretation. However, life-sized 3D printed models show great benefit in young residents (<1 year) regarding tumor localization and lead to a significantly more precise assessment of tumor extension.

## Data Availability

The raw data supporting the conclusions of this article will be made available by the authors, without undue reservation.
